# Achieving meaningful participation of people who use drugs and their peer organizations in a strategic research partnership

**DOI:** 10.1186/s12954-019-0306-6

**Published:** 2019-06-10

**Authors:** Graham Brown, Sione Crawford, Gari-Emma Perry, Jude Byrne, James Dunne, Daniel Reeders, Angela Corry, Jane Dicka, Hunter Morgan, Sam Jones

**Affiliations:** 1 Australian Research Centre in Sex, Health and Society, La Trobe University, Bundoora, VIC 3086 Australia; 2 Harm Reduction Victoria, PO Box 12720, A’Beckett Street, Melbourne, 8006 Australia; 3 Peer-Based Harm Reduction WA, PO Box 8003, Perth, WA 6849 Australia; 4 Australian Injecting & Illicit Drug Users League, GPO Box 1555, ACT, Canberra, 2601 Australia; 5 School of Regulation and Global Governance, Australian National University, ACT, Canberra, 2600 Australia

**Keywords:** People who use drugs, Peer organizations, Peer leadership, Meaningful involvement, Collaboration, Systems, W3 Project

## Abstract

**Background:**

Peer-led programs with people who use drugs (PWUD) have been a key characteristic of the harm reduction in many countries, including their involvement in research. However, peer involvement in research is often limited to recruitment, consultation, and reporting back, rather than a genuine collaboration in the priority setting, design, and conduct of research. PWUD peer organizations face ongoing challenges to demonstrate the depth of their knowledge of current and emerging issues within drug-using networks and the value of their peer insights for effective research and policy. The identification of benefits, barriers, and enablers for meaningful participation of PWUD in research has often been limited to methodological rather than system level factors.

**Methods:**

This paper draws on the experiences and findings of the What Works and Why (W3) Project, a 5-year collaborative study with peer organizations. The study drew on systems thinking methods to develop a framework to demonstrate the role of peer organizations within their community and policy systems. The study required peer staff and researchers to undertake the simultaneous role of drivers, participants, and analysts in the research. To identify the learnings in relation to meaningful participation of PWUD peer organizations in research, we drew together the insights and experiences of peer staff and researchers across the 5 years of the study

**Results:**

The W3 Project provided insights into the nuances of community-engaged research practice and the ongoing benefits, barriers, and enablers to the meaningful participation of PWUD and their peer organizations. These included system-level barriers and enablers beyond individual research projects or methodology. The capacity of research and peer organizations to maintain meaningful peer participation in research can be restricted or enhanced by the systems in which they are embedded.

**Conclusions:**

Recognizing peer organizations as active participants and drivers within community and policy systems can help clarify their unique and critical role in research. Achieving meaningful collaboration with PWUD peer organizations requires looking beyond good practice methods to the system-level factors with attention to the system-level benefits, barriers, and enablers.

## Background

Peer-based programs and organizations have been an integral part of health promotion and service delivery with people who use drugs (PWUD) for decades [1, 2]. They originated in Western Europe and North America in the late 1960s and evolved, developed, and expanded in response to emerging epidemics of HIV and hepatitis C in the 1980s and 1990s [3, 4]. At first, these programs were principally oriented towards the communities from which they emerged [3]. Later, as public health research and policymakers began to study and fund peer-based programs, peer organizations began to renegotiate their relationships with research projects and health systems [5–10].

PWUD peer organizations continuously adapt with their communities to new challenges and opportunities to improve the health, the well-being, and the rights of their communities [7, 11, 12]. PWUD have demonstrated that their organized peer-based response is a key component of effective national strategies for the public health response to HCV and HIV [2, 11, 13]. These peer organizations have expanded the dissemination of injecting equipment and social practices for safer injection, as well as disseminated knowledge that PWUD can use in everyday life to navigate the contextual pressures that weigh against safer use (including policing, criminalization, stigma, and discrimination) [11, 14–19]. However to achieve this, PWUD peer organizations are themselves vulnerable to, and must navigate, tensions between support for harm reduction-based health promotion and the stigmatization, prohibition, and criminalization of drug use which can undermine such support [20, 21].

The navigation of highly political contexts and circumstances has been a hallmark of these peer organizations [2, 22]. Their approach is often defined by their simultaneous engagement with community and policy systems and their ability to be nimble and flexible in responding to complex and politically volatile environments and contexts.

The participation of peer organizations within their communities, and their navigation of the political and health service contexts, provides them with knowledge of current and emerging issues within drug-using networks. The importance of this knowledge underpins the collaboration between PWUD peer organizations and researchers. PWUD peer workers have been found to assist in establishing more effective communication between communities of people who use drugs and research institutions, acting as a “bridge” in building or rebuilding trust and credibility and co-creating effective research priorities and methods [23].

However, the community and strategic insights peer organizations are able to provide can be undervalued by research, policy, and health services—and sometimes among peer organizations themselves [2, 10, 11, 24]. The engagement of PWUD in academic research has often been limited to consultation (for example, the inclusion on steering groups) and assistance with recruitment. This top-down, expert-driven mode of public health intervention that provoked the formation of peer organizations and peer-based programs and the “Nothing About Use Without Use” philosophy [1, 25] that informs their participation in policymaking applies equally to academic research seeking to recruit PWUD and represent their experiences. In this context, research that collaborates with PWUD peer organizations is a political as well as an evidence-based decision grounded in human rights principles.

In reflecting on the challenges and opportunities of research that engages with peer organizations and experience, this paper draws on the experiences and findings of the What Works and Why (W3) Project [26–28]. The project was a 5-year collaborative study with peer-led and community-based organizations that sought to develop a practice-based program theory and framework for peer programs, and then trial the application of this work within partner organizations [29]. The W3 Project provides insight into the nuances of community-engaged research practice and the ongoing benefits, barriers, and enablers of the meaningful involvement of PWUD peers and peer organizations in strategic research.

The paper first provides an overview of the W3 Project and its methods, with an emphasis on the ways peer organizations participated in driving the progress and evolution of the study through its different phases. We will then discuss the learnings from the study for achieving research that centers and enables the participation of peers and peer organizations at every stage of the research process. These learnings are drawn both from the experience of the study collaborators as well as findings the from the W3 Project research itself. These learnings are grouped under the headings of benefits, barriers, and enablers.

## W3 Project methods and involvement of PWUD peer organizations

The W3 Project partner organizations (Table 1) acknowledged the need to better understand how peer organizations contribute to Australia’s national strategies for HIV and HCV prevention and treatment. To achieve this understanding, we piloted the use of participatory methods for systems thinking and modeling, informed by the theory of complex adaptive systems [31–33]. Systems thinking helped us to identify and understand the complex relationships between all the moving parts of a community and policy system, and how they can generate emergent structures and effects. For example, the way the policy, health, justice, and research organizations enable, constrain, enhance, or resist PWUD peer organizations and their communities is an important part of understanding the role and impact of PWUD peer organizations in their system. The full methods of the W3 Project, and the development of the W3 Framework, are described in detail elsewhere [29]. Below, we have summarized these methods, with a focus on the role of peer participation and leadership throughout the study. We have added the partnership building prior to the project commencing and the subsequent trial and application of the W3 Framework, which have not been previously described [29].

**Table 1:** The W3 collaboration

W3 Project: understanding what works and why in peer-based and peer-led programs in HIV and hepatitis C Australian Federation of AIDS Organizations (the national peak body for the community-based response to HIV), Australian Injecting and Illicit Drug Users League (the national peak body for people who use drugs peer organizations), Harm Reduction Victoria (people who use drugs peer organization), Western Australian Substance Users Association (people who use drugs peer organization), Victorian AIDS Council (community and peer-based organization with services for gay and bisexual men, people who inject drugs, and people with HIV), Scarlet Alliance–Australian Sex Workers Association (national peer-based sex worker organization), National Association of People Living with HIV/AIDS (national people with HIV peer organization), Living Positive Victoria (people with HIV peer organization), Positive Life New South Wales (people with HIV peer organization), Queensland Positive People (people with HIV peer organization)	

In Australia, “community-based” and “peer-based” are the dominant organizational descriptors. These organizations were established by the communities most affected by HIV (and later HCV) from the mid-1980s, and their governance is based within their communities. Most of their limited funding comes from national and state governments with varying contracting conditions and caveats. Community-based organizations at a policy level are considered part of the “HIV partnership” alongside clinical services, research, and government. As with most countries, these relationships have waxed and waned over the past three decades [2, 30].

### Phase 1: Building the partnership

Prior to the W3 Project commencing, the first author had been conducting research into peer-based programs in health promotion for over a decade and had collaborated with many of the community and peer organizations who became part of the W3 Project. These collaborations identified the shared challenge of finding ways to demonstrate the quality and impact of peer-based programs and their role within the overall HIV and HCV response. Over a 12-month period, the first author hosted a series of meetings with the national, state, and territory peer and community organizations working in HIV and HCV. This resulted in collectively identifying the broad approach for the study and the ten organizations that were best placed to commit, to participate, and to collectively provide a broadly representative view in terms of communities of focus, organizational size, and geographic diversity (see Table 1). This initial phase included trialing a systems thinking workshop with senior management of the organizations to test elements of the proposed methods and build engagement. The collaboration successfully advocated for funding from the Australian Commonwealth Department of Health research investments (as part of the Australian national HIV and HCV strategies). The study was provided ethical approval by the La Trobe University Human Research Ethics Committee (Approval No: FHEC14/155).

### Phase 2: Participatory workshops to develop system maps

Over a 2-year period, we conducted a series of 18 highly participatory systems thinking workshops (described in more detail below) with ten HCV- and HIV-focused community- and peer-led organizations from across Australia who worked with communities of people who use drugs, gay men, sex workers, and people living with HIV (see Table 1 for the list of organizations). This included over 90 participants from the organizations. All the organizations included drug use within their programs and peer context. Three of the organizations were PWUD peer organizations (two state/territory and one national organization which advocates on behalf of state and territory member organizations at the national level), and the workshops included 23 peer staff from these organizations. We drew on the experience and perspectives of PWUD peer practitioners working in areas such as needle and syringe programs, outreach, community development, workshop facilitation, policy reform and leadership, management, and governance. The PWUD peer staff and volunteers varied in age (20s to 60s), gender (male, female, and non-binary), and drug use networks and experiences, with most having a history of using opiates and injecting drug use.

The aim of the workshops was to develop detailed system maps of how peer-led programs operate within their community and policy environments [24]. To do this, we drew on complex systems theory [31, 32, 34] and systems thinking methods (specifically soft systems methods as described by Checkland [35] and Williams and Hummelbrunner [36]) to elicit and draw together the mental models of the participants and their experience within peer organizations. The dynamics of the relationships between the peer programs, the peer organization, their communities, and the policy and health systems were identified, discussed, debated, and refined within the workshops. This meant the peer staff were simultaneously contributing insights and collaborating in analysis as the system maps were generated and refined over multiple days. Descriptions of the system maps developed by the study are available online [27].

### Phase 3: Analysis of system maps to identify common themes and functions

The full set of system maps [27] were analyzed to identify the key underlying functions and to develop a draft framework. This included negotiating within the partner organizations, an approach where we could conduct an iterative analysis that balanced the deep participation of the peer organization with the need to not overburden their investment in the study. The relationship built between the researchers and the peer organizations prior to and during the project meant the peer organizations identified a need for, and trusted, the research team to progress the analysis and move into a period of consultation and refinement of the framework. Each organization identified a peer worker with whom the research team could consult throughout the refinement process to generate an initial draft framework.

### Phase 4: Review and refinements by organizations locally and validated nationally

The draft W3 Framework developed through phase 3 was then subjected to review by additional peer organizations and stakeholders from across Australia, thanks to the support of national peak organizations (who advocate on behalf of and represent the voices of state and territory member organizations at the national level). For example, the W3 Framework was reviewed at a national peak PWUD peer organization meeting (involving an additional 11 participants from three state/jurisdictional PWUD peer organizations who had not been directly involved in the study). Incorporating these steps into the usual function of the sector was essential to reduce the burden on the peer organizations (such as requiring additional trips or consultations) and engaging with senior peer staff during a time when they were considering national and strategic issues. This also allowed the research to be framed within broader national policy discussions. This phase finalized the W3 Framework.

### Phase 5: Collaboratively develop indicators and tools

The W3 Project partnership was successful in accessing additional funds from the Australian Commonwealth Department of Health to apply the framework at a peer project and peer organization level. Over an 18-month period, the project used an action research approach [37] with two peer organizations (one peer-led drug user organization and one peer-led people with HIV organization) to develop, trial, and refine a broad range of quality and impact data collection tools that were usable, practical, and sustainable within the resources of the peer organizations. This included the development of key performance indicators and outcomes that demonstrated the role of peer organizations while also aligned with contracting requirements of funders. Peer staff were active participants in the development of the tools as well as the primary leads in trialing and refining the tools to be useful and sustainable. This required significant and ongoing time commitment from both peer and research staff. The first author spent on average half day per week in both peer organizations working with the peer staff as well as being part of the organization as a researcher in residence. The first author also participated in meetings between peer organizations and their funding agency as a critical friend and ally as the peer organizations applied the findings of the W3 Project into the scope of their funding agreements. This commitment from both the research staff and the peer organization staff was important to maintaining the trust and rapport between the researcher and the peer staff over a sustained period and minimizing the impact of changes in peer staff.

### Identifying lessons

To identify the learnings in relation to the participation of PWUD peer organizations in a sustained research study, we drew together the insights and experiences of peer staff and researchers across the 5 years of the study. This included detailed notes from the workshops in phase 2 to 4 [26, 28] and the resulting system map [27], as well as the field notes from the action research in phase 5. We also drew on formal interviews and informal discussions with peer management and staff of peer organizations conducted as part of assessing the impact of the W3 Project [38]. The learnings were then collated by author 1, 5, and 6 and validated by staff from peer organizations (authors 2, 3, 4, 7, 8, 9, and 10). We have grouped these learnings under the headings of benefits, barriers, and enablers to the involvement of PWUD peer organizations and peer leadership within research studies.

## Results: learnings on the involvement of PWUD peer organizations within research studies

Previous literature has identified good practice methods to the meaningful involvement of PWUD in policy, health services, and research (such as [1, 8, 19, 39–42]). However, much of this literature has focused on good practice engagement strategies and their benefits, with much less emphasis on the system level factors that may enhance or constrain meaningful involvement [6, 13]. Therefore, we have highlighted the learnings from the W3 Project which we felt were less represented in this literature.

### Benefits

#### Benefits to PWUD peer organizations and peer workers

The W3 Project provided the opportunity to bring together different perspectives and expertise from across the peer workforce and turn this into knowledge useful to the organizations. For example, the co-creation of the system maps in phase 2 meant that peer organizations were able to refine or reorient the framing of elements of the emerging system maps and the W3 Framework at all stages of development. This collaboration translated anecdotal and internal knowledge of peer organizations into a robust, trusted account and expressed this in language that could influence policymakers [31, 35, 43]. The sustained collaboration ensured the outcomes were directly relevant (conceptually and practically) to the work of peer organizations, creating meaningful research outcomes. Peer staff reported increased confidence and trust in interacting and negotiating with researchers, and peer organizations were better equipped to inform and influence research priorities and the broader research agenda.

#### Benefits to researchers and research centers

Investing in and strengthening the relationship with the collaborating peer organizations over a sustained period was an opportunity for the research center to demonstrate credibility and authenticity within the broader peer organization sector and their communities. For example, the relationships within the W3 Project collaboration opened up credibility with community networks not previously available to research staff. This has supported the research center’s other work, including strategic insights for identifying research priorities, recruitment of community participants into current studies, promotion and endorsement of study results, support in advocating for research investment, and the emergence of new collaborations. The sustained relationship and profile of the W3 Project also increased the research organization’s credibility in the broader policy and research sector as an informed ally of the PWUD community and peer organizations, and able to more effectively support the voice of PWUD in environments where PWUD peer leadership may still be stigmatized or less influential.

#### Benefits to the outcomes of the W3 Project

Participation of stakeholders was a central component to the methodology used within the W3 Project [29]. Drawing on peer insights and experience for both data and analysis ensured the understanding of the complex experiences of PWUD, and the cultural role of peer organizations within PWUD communities remained central to the study. For example, in phase 2 to 4, peer staff were able to identify interactions and connections within the system maps that were not evident without deep peer knowledge and expertise. When applying the W3 Framework to practice in phase 5, peer participation informed a balancing of research rigor, conceptual accuracy, and practical use grounded in the reality of peer organizations and their communities and funding environment. As the W3 Framework was a jointly developed and shared resource, other community and peer organizations were able to draw on the framework for their own initiatives (for examples see [44–46]) which provided additional insights into the usefulness of the framework. Supporting the peer organizations’ ownership within the study facilitated better translation of the research into real practice, with insights gained through that praxis then guiding further research.

### Barriers

#### The funding of peer organization services can be misaligned with their role in collaborating and advocating in research

The W3 Project found that peer-led health promotion is imbedded in the complex interactions between the peer programs they manage, the communities they participate in, and the policy and research environment within which they operate and collaborate [29]. Navigating this complex system was a key component of a peer organization’s successful influence and participation in policy and research. However, this system-level influence is generally not part of their service or program funding. We found that while peer organizations must navigate research and policy frameworks about community participation, peer leadership, and cross-sector partnership, peer projects are typically funded, monitored, and managed as purchaser/provider agreements with narrowly framed service provision outcomes. For example, maintaining a pool of strong and influential peer staff and volunteers is essential to any peer organization’s engagement with its communities. Less recognized is that this pool of peer staff and volunteers is also crucial to creating effective long-term partnerships with other organizations and essential to any meaningful participation in research. However, to recruit, support, and maintain such a pool of peers over time is an unrecognized (and typically unfunded) outcome of peer organizations managing a complex mix of discrete projects, each with narrowly constrained goals, often contracted by different funding sources. While this is not a new challenge, its impact on sustaining meaningful involvement in research is less well recognized. Peer leadership in research collaborations does not fit neatly into activity-based funding models that place emphasis exclusively on service delivery outputs. Further, there is a risk that building capacity in research advocacy may be interpreted as political advocacy, which many funding agreements implicitly or explicitly prohibit. These factors hinder the capacity of peer organizations to fulfill their policy participation role and to maintain their ability to influence or enhance research. The W3 Project was fortunate that the funders of the participating peer organizations demonstrated significant trust and flexibility in their commitment to peer organizations. There was an understanding of the interactive nature of the different peer projects, despite the difficulty in this being represented formally in funding agreements. This value placed on peer leadership by funders provided flexibility for the peer organizations to articulate research activity into the scope of funding agreements, allowing organizations to have some capacity to collaborate on research.

#### Peer leadership draws on communities already under pressure

A strength of peer organizations is their unique relationships within their communities [29]. This includes the ownership a community feels towards the peer organization, the drawing of staff and volunteers from the community, and the peer organization’s participation as a part of the community. However, this also adds a layer of complexity. Peer staff and volunteers are drawn from communities under pressure from marginalization, discrimination, criminalization, and higher rates of health complications, mental health challenges, and interaction with the legal/justice system. For example, the director of one of the peer organizations, the late Jenny Kelsall, was a strong leader within the peer community and the sector more generally. During the time of the W3 Project, Jenny became very ill for an extended period and passed away from liver cancer. The peer organization and its community experienced not only the loss of a highly respected and influential peer leader, but also the constant reminder that despite advances, their community was continuing to carry the burden of health issues and mortality due to HCV. While peer organizations have demonstrated incredible resilience and innovation [2, 7], these experiences nevertheless impact on the amount of flexibility there is across the peer staff and volunteer resources—both in time and emotional energy —to undertake reflexive practice, initiate new approaches, and meet the requirements of active participation in research processes [47]. For example, peer organizations are strong advocates for the co-design and action research approach to policy, health services, and research [1, 25]. However, co-design and action research (such as in the W3 Project) takes significant resources and emotional energy. It takes resilience to overcome the reluctance to invest limited resources in research after negative experiences from previous research. These experiences could be a mix of stigmatizing approaches, tokenistic participation, experiences of being “over researched” with repetitive studies, or having to orient and acclimatize each new researcher. While these are not necessarily new issues [25, 48, 49], the W3 Project found that at a peer staff or volunteer level, there is an emotional cost to playing the triple role of being collaborators, facilitators, and subjects of research. The workshop discussions in the W3 Project illustrated that identifying and then selecting which research is trustworthy and worth the “risk” and opportunity cost of being involved takes time and resources.

#### Political nature of peer programs with PWUD

PWUD peer organizations working in harm reduction may receive funding from policy outcomes with ideological rather than evidence-based origins (e.g., strategies to focus on harm reduction with a specific drug driven by media reporting rather than epidemiology [50]). The peer organizations are mindful in the way they adapt the implementation of these programs to remain relevant to their communities and their commitment to evidence-based practice, while navigating the policy agenda of funders. The system mapping work undertaken by the W3 Project required significant levels of trust between the peer organizations and the research team, and not all the nuance of the strategies and insights of navigating and influencing the complex policy and community systems could be shared outside of the study. The complexity of the nature of the work, the conflicting policy agendas, and the reasons or motivations for an adaptation cannot always be shared—but may be key to the outcome. From a research perspective, this can hinder full description of findings or sharing an understanding of the specificity of the context. While this was a challenge to be navigated in the development of the W3 Framework, we nevertheless found this “practice knowledge” of their complex system was present within these peer organizations. In other words, we found the peer staff were able to read between the lines as the nuance was not always explicitly stated in the research results.

#### Investment of time and resources does not guarantee timely benefits that are universally valued

For peer organizations, the benefits of investing in research are often not immediately visible or demonstrable, and so they can experience challenges to balance the possibility of future benefits against the need for visible short-term outcomes or immediate responses being demanded by their communities and funders. For example, the short-term benefits of participation in the W3 Project were not uniformly experienced across the peer organizations. Three PWUD peer organizations were involved in the development of the W3 Framework; however, the W3 Project only had the resources to work with one PWUD peer organization in the application of the framework to practice. The long-term benefits of the W3 Project for the broader peer sector are only now emerging and yet to be fully achieved.

For research centers, meaningful participation (as opposed to consultation) may be viewed as adding an additional layer of time and resources to the logistics of undertaking research. For example, the W3 Project methodology of participation through an iterative and reflexive study, and an emphasis on practice-based outcomes that were meaningful to peer organizations, limited the project’s capacity to translate the research quickly into multiple peer-reviewed journal papers. In the short term, meaningful collaboration can mean a focus on tailored outputs (such as community reports and briefings) and sector commitments (such as presentations, workshops, and training). This can result in delays in outputs that are more valued by academia (such as peer-reviewed publications). The broader research environment and structures that drive research funding do not always align with or value the time and resources required to maintain meaningful collaboration and share ownership of research direction with peer organizations.

Both PWUD peer organizations and research centers are constantly navigating shifting short- and long-term priorities. The opportunity cost of being involved in collaborative research is significant when resources are limited and timely benefits cannot be guaranteed. This needs to be recognized and incorporated into the planning of studies and the expectations of both research centers and peer organizations.

### Enablers

#### Flexibility in research funding, resources, and project management

The W3 Project was funded by the Australian Commonwealth Department of Health as part of the strategic research investments to support the Australian national HIV and HCV strategies. The nature of the fund allowed the researcher to have methodological flexibility to incorporate meaningful participation and to make a multiyear commitment to the peer organizations.

The flexibility was also essential at a project management level. For example, the study needed a broad range of peer staff to participate and so needed to adapt the methods while maintaining commitment to the broader methodology. This included adapting the approach of multiday workshops in a way which allowed staff to move in and out of the workshops without warning to sustain service delivery (such as delivering needle and syringe programs or meeting the needs of complex clients). Schedules also needed to remain very flexible, such as when workshops were deferred due to the peer organizations needing to respond quickly to an unexpected political development in the complex drug policy arena. The frequency and complexity of these events were not something experienced to the same extent by research centers and needed to be factored into research plans and methods.

#### Ongoing demonstration of two-way trust and commitment

The establishment and maintenance of trust and commitment between the research partners was crucial for the study to remain flexible. The researchers needed to trust in the expertise, experience, and perspective of the peer staff; while the peer staff needed to trust in the commitment and authenticity of the research team. This included the researchers sharing the research direction and analysis role and ownership of the outcomes. While this was underpinned by a collaboration contract between the peer organizations and the research center, it was also practical. For example, rather than the research team discussing an interpretation issue and coming to a shared position to then present to the peer organization collaborators, the peer staff were actively involved in these discussions and the disagreement between researchers became just part of a broader shared debate. Demonstrating an authorizing environment where ideas could be openly discussed and challenged meant the peer staff did not have to “overcome” a unified view of the researchers—they were part of forming the shared view from the beginning.

#### Visible valuing of peer participation and leadership to counter system-level stigma

Insights from peer organizations are often a source of real-time knowledge about emerging community issues or unintended consequences in rapidly changing environments. These insights need to be drawn together in a way that is useful for influencing policy and research. This can be an extremely high bar for often underfunded organizations navigating marginalized illicit drug cultures and stigma. Consistent with other studies [9, 11, 21, 51], the W3 Project system mapping found that in some policy and research environments, insights from PWUD peer programs were routinely stigmatized and dismissed due to a range of individual, institutionalized, and structural impediments to the involvement of PWUD. Peer organizations are aware of the power differences in the voices and opinions across the policy, health service, and community sectors. For peer organizations to have meaningful participation and influence in research and policy, they need an environment that values and enables them to have this role [29]. This requires more allies across the research, policy, and health service system with a commitment to share with the peer organization’s real-time insights about research and policy developments and visibly value and act on the peer organization’s advice regarding the implications for their communities. We found that an important enabler for achieving quality peer participation in research is to make more visible valuing of peer participation within the policy and research system more broadly. In the short term, collaboration between peer organizations and researchers can enhance peer influence through the leveraging of the researchers’ institutional position in the system, but in the longer term, ongoing visible collaboration enhances the recognition and valuing of the peer voice in and of itself, through the increased visibility of peers and peer organizations in the system.

## Discussion

Enhancing the meaningful involvement of PWUD and their peer organizations in research and policy has been an ongoing endeavor for decades [1] with literature discussing good practice as well as barriers and enablers [8, 25, 39–42, 52]. However much of this literature has focused on engagement strategies and research methodology, with much less emphasis on identifying system level factors that may enhance or constrain meaningful involvement [6, 13].

Drawing on the experiences and insights of a 5-year collaborative study into the system-level role of PWUD peer organizations, the W3 Project provided the opportunity to elicit insights and learnings regarding the system-level benefits, barriers, and enablers to meaningful involvement of PWUD peer organizations in research. The results suggest that PWUD peer organizations need the resources not only to demonstrate the validity of their connection and influence within their communities, but also to enhance and maintain their capacity to meet the demands of implementing and sustaining meaningful participation across research and policy. The results also illustrate that while research programs need the resources and flexibility to sustain meaningful relationships with peer organizations, this is not enough. To increase the support for and capacity of sustained PWUD peer participation, research organizations also need to be visible in their valuing of PWUD peer organizations and their participation in the broader research, government, and health services sector.

If research is to achieve meaningful and useful collaboration with peer organizations, then the issues involved need to be recognized as more than issues of methodology. We found that the capacity of research and peer organizations to sustain a commitment to meaningful peer participation in research can be restricted or enhanced by the complex systems in which they are embedded, and so system-level issues must be tackled as well.

The W3 Project, in phase 5, worked towards the development of indicators for the role and impact of peer organizations in community and policy systems [29]. Identifying system-level indicators to demonstrate the outcomes of meaningful participation of PWUD peer organizations in research, with attention to the system-level benefits, barriers, and enablers, may also be of benefit.

The lessons we have identified in this study have limitations in their generalizability. We only drew from the experiences of peer staff and researchers who participated in the W3 Project which was only conducted in Australia. The benefits, barriers, and enablers we identified may not be transferable to other contexts or countries as drug policy, legislative, and research environments vary significantly.

## Conclusions

The W3 Project applied systems thinking to understanding the role of peer organizations in a public health response and the influence of peer-led programs within their communities and policy systems. Through this, the multiyear collaborative study provided insights into achieving meaningful participation of PWUD and their peer organizations.

Recognizing peer organizations as active participants and drivers within community and policy systems can help demonstrate more clearly their unique and critical role in research. Achieving and maintaining meaningful collaboration with PWUD peer organizations requires looking beyond good practice methods to the system-level factors with attention to the system-level benefits, barriers, and enablers.

## Data Availability

Detailed methodology, system map descriptions, and other materials generated during the W3 Project are available online from the W3 website (www.w3project.org.au) and cited throughout the paper with specific URL in the references.
